# Supplementary material for: Comparison of Microglial Morphology and Function in Primary Cerebellar Cell Cultures on Collagen and Collagen-Mimetic Hydrogels
Source: Biomedicines. 2022 Apr 29;10(5):1023. doi: 10.3390/biomedicines10051023 (PMC9139096; doi:10.3390/biomedicines10051023)
Supplement: Supplementary file 1 [file biomedicines-10-01023-s001.zip › Supplementary Figure S1.pdf]

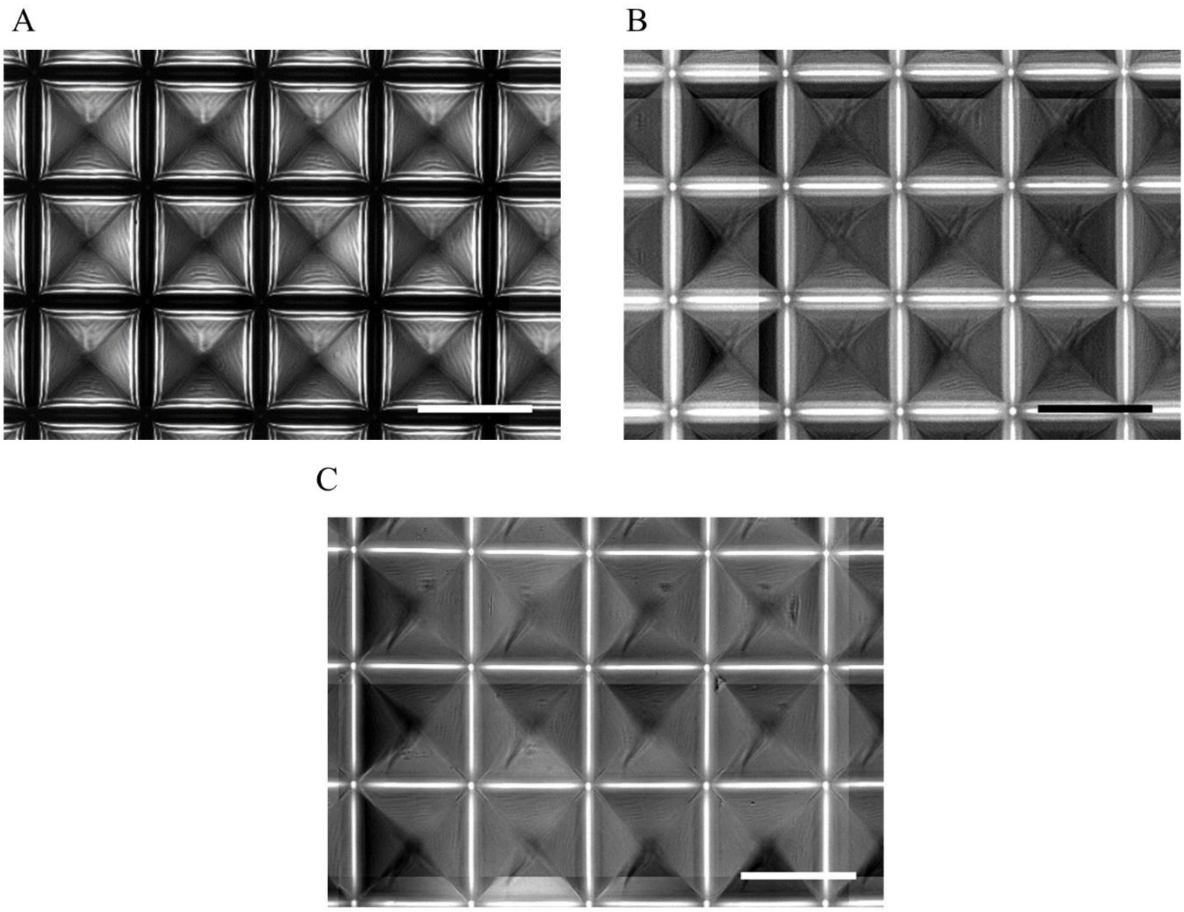

Supplementary Figure S1. Representative stitched brightfield microscopy images of AggreWell™ 400 (A), PDMS replica (B), and CLP-PEG hydrogel microwells (C). Images were obtained in the air environment using an Olympus BX51 upright microscope (Olympus, Tokyo, Japan) equipped with a 10×, NA 0.3 air objective and a Peltier-cooled Fview II CCD camera (Olympus Soft Imaging Solutions GmbH, Münster, Germany) and stitched using analySIS software (Olympus Soft Imaging Solutions GmbH, Münster, Germany). The scale bar is 400 μm.
